# Visualizing traumatic stress-induced structural plasticity in a medial amygdala pathway using mGRASP

**DOI:** 10.3389/fnmol.2023.1313635

**Published:** 2023-11-30

**Authors:** Caitlyn J. Bartsch, Jessica T. Jacobs, Nooshin Mojahed, Elana Qasem, Molly Smith, Oliver Caldwell, Sophia Aaflaq, Jacob C. Nordman

**Affiliations:** Department of Physiology, Southern Illinois University School of Medicine, Carbondale, IL, United States

**Keywords:** medial amygdala, ventromedial hypothalamus, traumatic stress, mGRASP, structural plasticity, NMDARs

## Abstract

Traumatic stress has been shown to contribute to persistent behavioral changes, yet the underlying neural pathways are not fully explored. Structural plasticity, a form of long-lasting neural adaptability, offers a plausible mechanism. To scrutinize this, we used the mGRASP imaging technique to visualize synaptic modifications in a pathway formed between neurons of the posterior ventral segment of the medial amygdala and ventrolateral segment of the ventromedial hypothalamus (MeApv-VmHvl), areas we previously showed to be involved in stress-induced excessive aggression. We subjected mice (7–8 weeks of age) to acute stress through foot shocks, a reliable and reproducible form of traumatic stress, and compared synaptic changes to control animals. Our data revealed an increase in synapse formation within the MeApv-VmHvl pathway post-stress as evidenced by an increase in mGRASP puncta and area. Chemogenetic inhibition of CaMKIIα-expressing neurons in the MeApv during the stressor led to reduced synapse formation, suggesting that the structural changes were driven by excitatory activity. To elucidate the molecular mechanisms, we administered the NMDAR antagonist MK-801, which effectively blocked the stress-induced synaptic changes. These findings suggest a strong link between traumatic stress and enduring structural changes in an MeApv-VmHvl neural pathway. Furthermore, our data point to NMDAR-dependent mechanisms as key contributors to these synaptic changes. This structural plasticity could offer insights into persistent behavioral consequences of traumatic stress, such as symptoms of PTSD and social deficits.

## Highlights

– The medial amygdala (MeA) regulates behavioral responses to traumatic stress.
– Using mGRASP, we observe that foot shock increases synapse formation in a MeA pathway.
– Inhibiting excitatory MeA neurons and blocking NMDARs suppressed the stress-induced synaptic changes.
– The structural changes in this MeA pathway may reveal important implications for PTSD.

## 1 Introduction

The increasing prevalence of mental health conditions like major depression, PTSD, anxiety disorders, and bipolar disorder presents a significant global health challenge. As of 2021, nearly one in five adults in the United States were diagnosed with a mental illness, and depressive disorders are a leading cause of disability (National Institutes of Health and the Substance Abuse and Mental Health Services Administration). Current treatment modalities, including pharmacotherapy and psychotherapy, have remained largely unchanged for decades, and often come with limitations such as adverse side effects and low remission rates (Sachsse et al., 2006; American Psychiatric Association [APA], 2013; Watson et al., 2016; Bartsch and Nordman, 2022). These issues underscore the urgent need for innovative research methods to enhance our understanding and treatment of psychiatric conditions.

Traumatic stress can lead to enduring changes in behavior, including increased aggression, depression, anxiety, and other mental health disorders (Connor and Davidson, 2001; American Psychiatric Association [APA], 2013; Taft et al., 2017). These effects are possibly attributed to the brain’s inherent plasticity, especially during postnatal development (Nemeroff et al., 2006; Sherin and Nemeroff, 2011; Trickey et al., 2012; Lewis et al., 2019). Previous work from our lab has demonstrated that traumatic stress can potentiate synapses implicated in aggressive behavior, focusing on the neural circuits originating from the posterior ventral segment of the medial amygdala (MeApv) that project to the ventrolateral segment of the ventromedial hypothalamus (VmHvl) (Nordman et al., 2020a,b).

The MeA is a key area in violent aggression (Nelson, 2006; Nelson and Trainor, 2007; Hong et al., 2014; Nordman et al., 2020a,b), and is a successful target for neurosurgical interventions to treat intractable escalated aggression (Mpakopoulou et al., 2008). The MeApv primarily contains glutamatergic neurons that participate in experience-dependent aggression (Nordman et al., 2020a,b). Notably, these excitatory MeApv neurons send dense projections to the VmH, a canonical member of the aggression circuit that plays a significant role in behavioral responses to traumatic stress (Shaikh et al., 1986; Silva et al., 2013; Masugi-Tokita et al., 2016; Hashikawa et al., 2017; Nordman et al., 2020a,b).

To further explore the neural mechanisms underlying maladaptive responses to traumatic stress, we employed a PTSD stress model devised for this purpose (Nordman et al., 2020a,b, 2022; Bartsch et al., 2023). This model integrates chronic social isolation during early adolescence with acute traumatic stress in late adolescence, offering a multidimensional perspective that more closely resembles complex real-world trauma scenarios (Vlachos et al., 2020). In addition, this study employs a highly precise synaptic imaging method known as mGRASP (mammalian GFP Reconstitution Across Synaptic Partners) to examine synaptogenesis—or the formation of new synapses—within an excitatory MeApv-VmHvl pathway (Kim et al., 2011; Feng et al., 2012, 2014). This imaging technique uniquely enables us to visualize and quantify specific synaptic changes at a level of detail that is currently unparalleled.

Our results reveal a significant increase in synapse number in the MeApv-VmHvl pathway following traumatic stress, driven primarily by excitatory synaptic activity as evidenced by chemogenetic techniques. Moreover, these changes are mediated by *N*-methyl-D-aspartate receptor (NMDAR)-dependent mechanisms, as shown through the administration of the NMDAR antagonist MK-801. These findings not only deepen our understanding of the neural circuits affected by traumatic stress but also offer potential molecular targets for future therapeutic interventions.

Given the clinical implications of these neural changes, our insights could be invaluable for developing targeted treatments for mental health conditions induced by traumatic stress. These could include more effective pharmacological agents that modulate synaptic plasticity and connectivity, ultimately contributing to more efficacious and personalized treatment options for those suffering from trauma-induced behavioral disorders.

## 2 Materials and methods

### 2.1 Animals

All animal protocols were approved by the Animal Care and Use Committee of Southern Illinois University School of Medicine. All C57BL/6 mice used in this study were purchased from Charles River Laboratories and housed under a reverse 12-h light (8 pm–8 am)/dark (8 am–8 pm) cycle with *ad libitum* access to water and food. Starting at 3–4 weeks of age, mice were socially isolated for 4 weeks before testing (Nordman et al., 2020a,b, 2022; Bartsch et al., 2023). Surgical procedures, outlined below, were performed at 5–6 weeks of age. Male mice were used for all experiments due to sex-specific effects of the MeA on aggression (Unger et al., 2015).

### 2.2 Surgical procedures

Five-six–week old C57BL/6 male mice were anaesthetized with isoflurane (3% for induction and 1% for maintenance) and then placed onto a stereotaxic frame (David Kopf Instruments). Unilateral or bilateral craniotomy was made and 250–500 nl of a viral suspension was injected into a region of interest using a 5 μl gas-tight Hamilton Syringe (33-gauge) at a rate of 25 nl/min (Supplementary Figure 1). After injection, the needle was left in place for an additional 10 min and then slowly withdrawn. The adeno-associated viruses (AAV) used were pAAV2-CAG-pre-mGRASP-mCerulean (pre-GRASP, Addgene #34910, 2.3 × 10^13^ vg/mL), pAAV2-CAG-Post-mGRASP-2A-dTomato (post-GRASP, Addgene #34912), and pAAV9-CaMKIIa-hM4D(Gi)-mCherry [hM4D(Gi), Addgene #50477, 2.1 × 10^13^ vg/mL]. A total of 250 nl of each virus was injected into the following coordinates: MeApv, AP = −2.1 mm, ML = 1.5, DV = −5.25 mm; VmHvl, AP = −2.1 mm, ML = 0.6 mm, DV = −5.8 mm (Nordman et al., 2020a,b; Nordman and Li, 2020). pAAV9-CaMKIIa-hM4D(Gi)-mCherry was injected bilaterally. Skin was sealed using Vetbond. Keto fluids were administered for 3–5 days post-surgery. Mice were allowed to recover for 2 weeks.

### 2.3 Traumatic stress-induction and drug injections

After 4 weeks of social isolation in a reverse light cycle, surgical animals were transferred from their housing room to a darkened behavior room and left alone to acclimate for 1 h prior to stress induction. They were then placed into a fear conditioning chamber within a sound-attenuating cubicle (Med Associates). After a 3 min exploration period, 15 non-contingent electric foot shocks (0.4 mA, 1 s in duration) were administered through an electrified grate at random intervals (240–480 s) over 90 min (Nordman et al., 2020a,b, 2022; Bartsch et al., 2023). For chemogenetic and pharmacology experiments, mice were injected with saline (vehicle), clozapine N-oxide (CNO), or MK-801 30 min before foot shock. Mice were then transferred from their home cages by gently picking the animals up by the base of their tails, placing them into a cup, and then putting them inside the fear conditioning chamber. Care was taken to avoid degloving. Mice were returned to their home cages using the same method.

### 2.4 Pharmacology

MK-801 (150 μg/kg, Tocris) (Newman et al., 2012, 2018; Nordman et al., 2020a,^2022^) and clozapine-N-oxide (1 mg/kg CNO, Sigma) were dissolved in 0.9% saline (Chang and Gean, 2019). Seven-eight-week-old mice were injected intraperitoneally (IP) using a 27-gauge needle 30 min before being placed in the fear conditioning chamber. A total of 0.9% saline was used as a vehicle control.

### 2.5 Imaging and analysis

We utilized a modified protocol to analyze mGRASP puncta (Feng et al., 2012). In brief, we captured z-stack images of 4–7 randomly selected dendrites from 3 30 μm-thick brain slices encompassing both the MeApv and VmHvl regions with a Leica DM5500 Q confocal microscope, using an PlanFL PH2 10x/0.30 (RWD) and ACS APO 63x/1,30 oil objective lens (model 11507900) at 2x magnification (Supplementary Figure 1). The acquired images were then processed with ImageJ software to generate a 3D representation of each dendrite alongside its associated cell body. For puncta detection, we employed a thresholding technique followed by watersheding and mean shifting for image segmentation. Only mGRASP and spine puncta that were at least 2 pixels × 2 pixels in size after thresholding and watersheding were counted.

Within ImageJ, we measured dendritic lengths, puncta count, and area. For consistent quantification, puncta were counted on a single dTomato + proximal and distal dendrite from one cell. Results were then normalized to a 10 μm length segment—a standard previously set by our lab for quantifying dendritic protrusions (Jia et al., 2014). Only dendrites expressing at least one 2 × 2 pixel minimum mGRASP puncta were counted. Proximal was defined as a dendrite 0–50 μm from the soma, while distal was defined as a branched dendrite greater than 50 μm from the soma (Perez-Rando et al., 2017).

### 2.6 Statistical analysis

**FIGURE 1:**
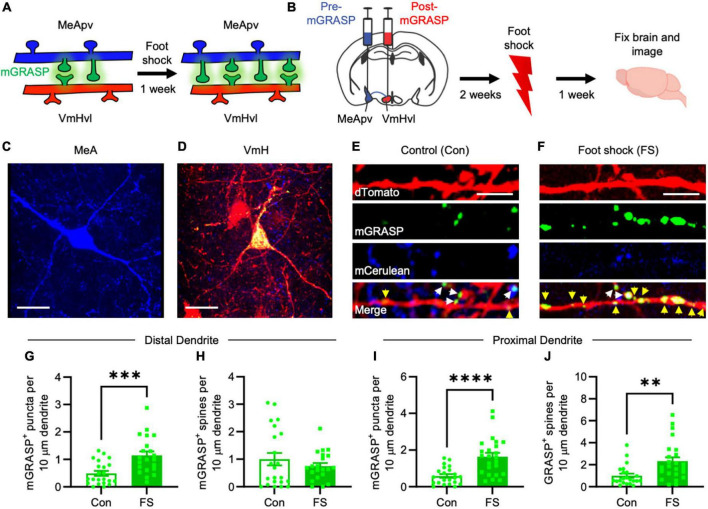
More mGRASP puncta were found on VmHvl dendrites of foot shocked mice compared to non-shocked controls, suggestive of synaptogenesis in the MeApv-VmHvl pathway. **(A)** Hypothesized effect of foot shock on structural plasticity within an MeApv-VmHvl pathway, measured using mGRASP. **(B)** Experimental schedule for inducing traumatic stress via foot shock. Representative images of MeApv cells expressing mCerulean **(C)** and VmHvl cells expressing dTomato and mGRASP **(D)**. mCerulean in **(D)** are MeApv axons. Scale bars = 25 mm. Representative images of dendrites expressing mGRASP 7 days after foot shock **(F)** or control condition [in shock box but with no foot shocks, **(E)**]. Yellow arrows indicated shaft synapses and white arrows indicate spine synapses. Scale bars = 5 mm. Quantification of mGRASP puncta on the shafts **(G,I)** or spines **(H,J)** of distal dendrites (*n* = 22 dendrites from 3 control mice and 21 dendrites from 4 FS mice) or proximal dendrites (22 dendrites from 3 control mice and 24 dendrites from 4 FS mice), normalized to 10 mm length segments. Mean ± SEM, **p < 0.01, ***p < 0.001, ***p < 0.0001.

**FIGURE 2:**
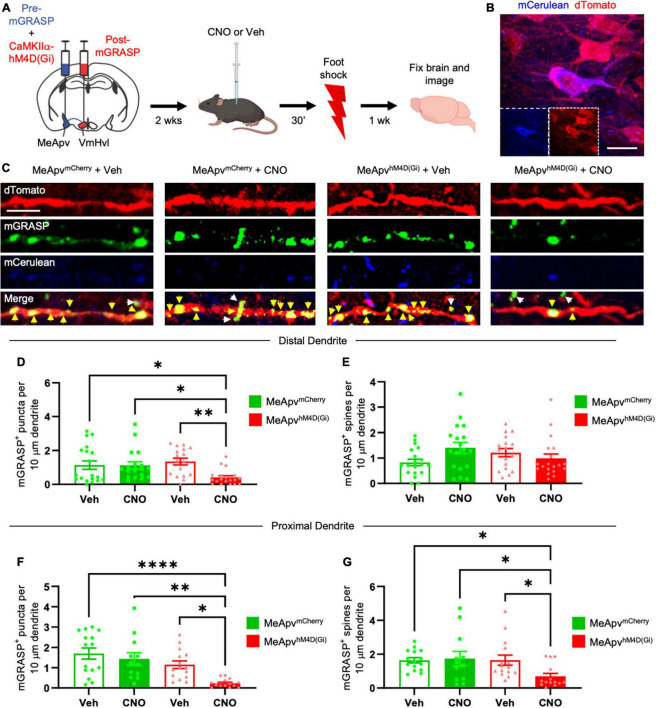
Chemogenetic inhibition of excitatory MeApv neurons suppresses foot shock-induced increases in mGRASP puncta on VmHvl dendritic shafts. **(A)** Experimental schedule for chemogenetically inhibiting excitatory MeApv neurons before foot shock. Mice received injections of pre-mGRASP and hM4D(Gi) (the inhibitory DREADD receptor) into the MeApv, and post mGRASP into the VmHvl. **(B)** Representative images of an MeApv neuron expressing pre-mGRASP and hM4D(Gi) (conjugated to mCherry). Scale bar = 25 mm. **(C)** Representative images of mGRASP on dTomato-expressing VmHvl dendrites 7 days post-foot shock for all four conditions: mice that were injected with vehicle and expressed mCherry control virus in the MeApv (MeApv*^mCherry^* + Veh); mice that were injected with CNO and expressed mCherry in the MeApv (MeApv*^mCherry^* + CNO); mice that were injected with vehicle and expressed hM4D(Gi) in the MeApv (MeApv*^hM4D^* + Veh); mice that were injected with CNO and expressed hM4D(Gi) in the MeApv (MeApv*^hM4D^* + CNO). Yellow arrows indicate shaft synapses and white arrows indicate spine synapses. Scale bar = 5 mm. Quantification of mGRASP puncta on the shafts **(D,F)** or spines **(E,G)** of distal dendrites (*n* = 19 dendrites from 3 MeApv*^mCherry^* + Veh mice, 18 dendrites from 3 MeApv MeApv*^mCherry^* + CNO mice, 17 dendrites from 3 MeApv*^hM4D^* + Veh mice, and 19 dendrites from 3 MeApv*^hM4D^* + CNO mice) or proximal dendrites (*n* = 15 dendrites from 3 MeApv MeApv*^mCherry^* + Veh mice, 12 dendrites from 3 MeApv MeApv*^mCherry^* + CNO mice, 15 dendrites from 3 MeApv*^hM4D^* + Veh mice, and 14 dendrites from 3 MeApv*^hM4D^* + CNO mice) from all conditions, normalized to 10 mm length segments. Mean ± SEM, *p < 0.05, **p < 0.01, ****p < 0.0001.

**FIGURE 3:**
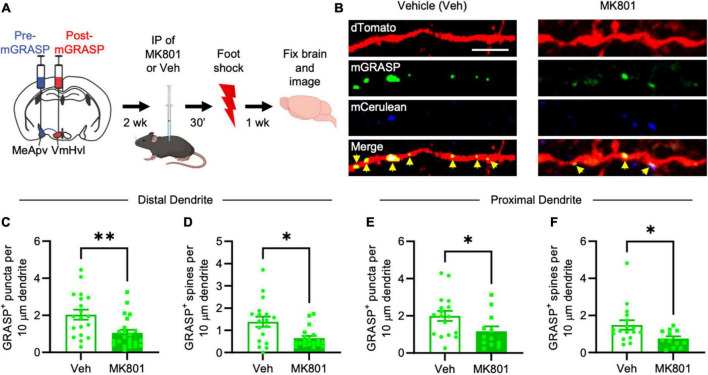
MK-801 suppresses foot shock-induced long-lasting increases in mGRASP puncta at MeApv-VmHvl synapses. **(A)** Experimental schedule for inhibiting NMDARs with MK-801 before foot shock. **(B)** Representative images of dendrites expressing mGRASP 7 days after IP injections of MK-801 or vehicle followed by foot shock. Yellow arrows indicated shaft synapses. Scale bar = 5 mm. Quantification of mGRASP puncta on the shafts **(C,E)** or spines **(D,F)** of distal dendrites (*n* = 19 dendrites from 4 vehicle-treated mice and 23 dendrites from 4 MK-801-treated mice) and proximal dendrites (*n* = 17 dendrites from 4 vehicle-treated mice and 12 dendrites from 3 MK-801-treated mice), normalized to 10 mm length segments. Mean ± SEM, *p < 0.05, **p < 0.01.

Detailed statistics can be found in Table 1. All data were presented as mean ± SEM. Prism software was used for statistical analysis. Sample sizes were determined using power analyses, with an alpha = 0.05 and a beta = 0.8. Mann Whitney tests were used for Figures 1G–J, 3C–F and Supplementary Figures 1D, O, 2, 4, as a result of normality tests. Two-way ANOVAs were used for Figures 2D–G and Supplementary Figures 2K, 3, comparing mice that expressed Designer Receptors Activated Only by Designer Drugs (DREADD) receptors to mice that expressed mCherry control virus, each group of which received IP injections of CNO or vehicle. Tukey’s tests were used for multiple comparisons to identify groups that were significantly different. *p* < 0.05 was considered significant and all tests were two-tailed with significance is denoted by asterisks.

**TABLE 1:** Statistics table for all data in the manuscript.

Data	Method	*N* (brain slices)	F or U stat	*p*-Value	*Post-hoc* test
Figure 1G	Mann-Whitney	22, 21	*U* = 91	0.0004	
Figure 1H	Mann-Whitney	22, 21	*U* = 228	0.9471	
Figure 1I	Mann-Whitney	22, 24	*U* = 84	<0.0001	
Figure 1J	Mann-Whitney	22, 24	*U* = 120	0.0012	
Figure 2D	Two-way ANOVA	19, 18, 17, 19	*F*_(3, 51)_ = 5.901	0.0015	Tukey’s
Figure 2E	Two-way ANOVA	19, 18, 17, 19	*F*_(3, 51)_ = 2.493	0.0704	Tukey’s
Figure 2F	Two-way ANOVA	15, 12, 15, 14	*F*_(3, 38)_ = 9.362	<0.0001	Tukey’s
Figure 2G	Two-way ANOVA	15, 12, 15, 14	*F*_(3, 38)_ = 4.168	0.0120	Tukey’s
Figure 3C	Mann-Whitney	19, 23	*U* = 104	0.0032	
Figure 3D	Mann-Whitney	19, 23	*U* = 97	0.0242	
Figure 3E	Mann-Whitney	17, 12	*U* = 54	0.0339	
Figure 3F	Mann-Whitney	17, 12	*U* = 51	0.0236	
Supplementary Figure 1D	Mann-Whitney	43, 44	*U* = 330	<0.0001	
Supplementary Figure 1K	Two-way ANOVA	25, 22, 30, 33	*F*_(3, 74)_ = 16.54	*P* < 0.0001	Tukey’s
Supplementary Figure 1O	Mann-Whitney	33, 33	*U* = 356	0.0151	
Supplementary Figure 2A	Mann-Whitney	25, 28	*U* = 187.5	0.0033	
Supplementary Figure 2B	Mann-Whitney	21, 21	*U* = 172	0.2299	
Supplementary Figure 2C	Mann-Whitney	21, 23	*U* = 234.5	0.8754	
Supplementary Figure 2D	Mann-Whitney	15, 21	*U* = 99	0.0622	
Supplementary Figure 2E	Mann-Whitney	28, 30	*U* = 243	0.0053	
Supplementary Figure 2F	Mann-Whitney	16, 21	*U* = 110	0.0778	
Supplementary Figure 2G	Mann-Whitney	26, 25	*U* = 290.5	0.5219	
Supplementary Figure 2H	Mann-Whitney	19, 22	*U* = 204.5	0.9125	
Supplementary Figure 3A	Two-way ANOVA	19, 18, 17, 19	*F*_(3, 51)_ = 2.141	0.1065	Tukey’s
Supplementary Figure 3B	Two-way ANOVA	15, 12, 15, 14	*F*_(3, 38)_ = 2.115	0.1144	Tukey’s
Supplementary Figure 4A	Mann-Whitney	19, 23	*U* = 206	0.7590	
Supplementary Figure 4B	Mann-Whitney	17, 12	*U* = 100	0.9479	

## 3 Results

### 3.1 Foot shock induces long-lasting structural plasticity in a MeApv-VmHvl pathway

Utilizing the mGRASP system, we sought to explore synaptogenesis within a MeApv-VmHvl pathway (Figure 1A). Initial injections were carried out in 5–6-week-old male mice with pAAV-CAG-pre-mGRASP-mCerulean into the MeApv and pAAV-CAG-post-mGRASP-2A-dTomato into the VmHvl (Figure 1B and Supplementary Figures 1A–C). Three weeks later, mice were euthanized, and the brains extracted to examine mGRASP expression within the VmHvl. We observed extensive mCerulean expression in MeApv boutons and axons (Figure 1C and Supplementary Figures 1A, B) and dTomato and GFP on VmHvl dendritic shafts, spines, and the soma (Figure 1D and Supplementary Figure 1C), validating our use of this technique.

For traumatic stress induction, separate groups of 5–6-week-old mice were delivered injections of the mGRASP fragments and then socially isolated for 2 weeks. Mice were then placed into a fear conditioning chamber where they received 15 non-contingent foot shocks over 90 min (FS, 4 mice, Figure 1B), a protocol we have developed and used extensively (Nordman et al., 2020a,b, 2022; Bartsch et al., 2023). Control mice (Con, 3 mice) were left in the shock box for the same period of time but received no foot shocks.

Seven days later, brains were harvested and then sectioned. Using confocal microscopy, we observed a significant increase in the number and size of mGRASP puncta on both proximal and distal VmHvl dendritic shafts (Figures 1E–G, *P* = 0.0004; Figure 1I, *P* < 0.0001, Supplementary Figure 2B, *P* = 0.0033, Supplementary Figure 2E, *P* = 0.0053). Similar effects were found when we normalized mGRASP puncta counts to mCerulean expression in the MeApv (Supplementary Figure 1D, *P* < 0.0001). Interestingly, foot shock had a significant effect on proximal, but not distal, mGRASP + spines (Figure 1H, *P* = 0.9471; Figure 1J, *P* = 0.0012). No significant effects were observed for total number of distal or proximal VmHvl spines (Supplementary Figure 2B, *P* = 0.2299; Supplementary Figure 2F, *P* = 0.0778), area of mushroom spines (Supplementary Figure 2C, *P* = 0.8754; Supplementary Figure 2G, *P* = 0.5219) and area of mGRASP on mushroom spines (Supplementary Figure 2D, *P* = 0.0622; Supplementary Figure 2H, *P* = 0.9125). These results suggest that foot shock predominantly effects shaft synapses and proximal spines formed between the MeApv and VmHvl.

Broadly, these findings substantiate our hypothesis that traumatic stress fosters structural plasticity changes in the MeApv-VmHvl pathway.

### 3.2 Excitatory MeApv signaling drives structural plasticity in the MeApv-VmHvl pathway after foot shock

We previously found that excitatory input from the MeApv drives foot shock-induced aggression through synaptic plasticity at the VmHvl (Nordman et al., 2020a,b). To probe the role of excitatory input from the MeApv in the observed structural plasticity, we employed a chemogenetic approach alongside the mGRASP technique (Roth, 2016; Chang and Gean, 2019). Mice received AAV injections into the MeApv of a mixture of pre-mGRASP and either the chemogenetic inhibitory DREADD receptor, hM4D(Gi), or mCherry control virus. hM4D(Gi) and mCherry were under the control of the CaMKIIα promoter, which restricts expression of the transgene to excitatory neurons in the MeApv (Nordman et al., 2020a,b). We previously confirmed the efficacy of the CaMKIIa promoter in labeling glutamatergic neurons in this region by showing that >85% of vGlut2 + neurons expressing GFP were positive for CaMKIIα and none of the CaMKIIα + neurons colocalized with the inhibitory markers calbindin, calretinin, somatostatin, or parvalbumin (Nordman et al., 2020a). vGlut2 is widely expressed by glutamatergic neurons throughout the MeApv (Choi et al., 2005; Cheong et al., 2015; Ishii et al., 2017).

To ensure that the pre-mGRASP + neurons expressed the DREADD receptor, we examined mCerulean cells for the presence of mCherry. Pre-mGRASP + MeApv cells were positive for >95% of mCherry in vehicle-treated mice (*n* = 3 mice), >93% of mCherry in CNO-treated mice (*n* = 3 mice), >88% of hM4D(Gi) in vehicle-treated mice (*n* = 3 mice), and >91% of hM4D(Gi) in CNO-treated mice (*n* = 3 mice). These results strongly suggest that the observed pre-mGRASP puncta on MeApv neurons were excitatory (Figures 2B, C and Supplementary Figures 1E, F, H, I) and that the MeApv-VmHvl synapses were glutamatergic. Mice were then socially isolated for 2 weeks.

On the day of traumatic stress induction, mice received IP injections of the hM4D(Gi) ligand CNO [25 μg/ml, *n* = 4 hM4D(Gi)-expressing mice and 3 mCherry control-expressing mice] (Roth, 2016; Chang and Gean, 2019) or vehicle [*n* = 4 hM4D(Gi)-expressing mice and 3 mCherry control-expressing mice] 30 min prior to foot shocks (Figure 2A). IP injections of CNO were used because implantation of cannulae and direct drug infusion could damage the VmHvl, making it difficult to properly detect the mGRASP signal. Seven days later, brain samples were analyzed for mGRASP puncta, comparing CNO and vehicle-treated mice.

We observed significantly fewer mGRASP puncta on both the distal and proximal dendritic shafts of VmHvl neurons in the hM4D(Gi)-expressing mice injected with CNO compared to the control conditions (Figures 2C, D, *P* = 0.0015; Figure 2F, *P* < 0.0001). Similar results were observed when normalized to mCerulean expression (Supplementary Figure 1K, *P* < 0.0001). Chemogenetic inhibition suppressed the increase in mGRASP puncta on proximal, but not distal, spines (Figure 2E, *P* = 0.0704; Figure 2G, *P* < 0.0001). No significant change was observed in the total number of VmHvl spines between conditions (Supplementary Figure 3A, *P* = 0.1065; Supplementary Figure 3B, *P* = 0.1144).

These findings strongly suggest that excitatory neurotransmission from MeApv neurons is instrumental in mediating structural plasticity in the MeApv-VmHvl pathway.

### 3.3 NMDARs mediate structural plasticity in the MeApv-VmHvl pathway

We previously reported that attack experience potentiates MeApv-VmHvl synapses and heightens aggression via an NMDAR-dependent mechanism (Nordman et al., 2020a). Recently, we found that the non-competitive NMDAR antagonist MK-801 suppresses foot shock-induced long-lasting aggression in socially isolated mice (Nordman et al., 2022). Similar findings were reported by another group (Chang et al., 2015). Given that attack-experience and traumatic stress promote aggression through potentiation of the same MeApv pathways, it stands to reason that traumatic stress induces synaptogenesis in the MeApv-VmHvl pathway through an NMDAR-dependent mechanism as well.

To test this hypothesis, we again injected the pre- and post-mGRASP fragments into the MeApv and VmHvl, respectively. After 2 weeks of social isolation, mice received IP injections of 3.75 μg/ml MK-801 (*n* = 4 mice) diluted in 0.9% saline or 0.9% saline alone (Veh, *n* = 4 mice) 30 min before foot shock (Figure 3A) (Petralia et al., 2007; Zhang et al., 2017; Nordman et al., 2020a,^2022^). This concentration does not affect mobility nor act as an antidepressant (Nordman et al., 2020a; Bartsch et al., 2023). As before, IP injections of MK-801 were used because cannulae and direct infusion could damage the VmHvl, making it difficult to properly detect the mGRASP signal.

Brain slices containing the VmHvl were harvested 7 days after foot shock and then examined for a difference in the number of GFP puncta between MK-801 and vehicle-injected mice. Our findings reveal a significant reduction of mGRASP puncta on the spines and dendritic shafts of VmHvl neurons in MK-801-treated mice compared to vehicle injected controls (Figures 3B, C, *P* = 0.0032; Figure 3D, *P* = 0.0242; Figure 3E, *P* = 0.0339; Figure 3F, *P* = 0.0236). Similar results were observed when normalized to mCerulean expression (Supplementary Figure 1O, *P* = 0.0151). No effects were found on overall spine number (Supplementary Figure 4A, *P* = 0.7590; Supplementary Figure 4B, *P* = 0.9479). These results suggest that traumatic stress-induced structural plasticity in the MeApv-VmHvl pathway is largely NMDAR-dependent.

In summary, our results highlight the enduring impact of traumatic stress on structural plasticity within the MeApv-VmHvl pathway, primarily affecting synapse number on the dendritic shaft. The excitatory neurons in the MeApv appear to play a pivotal role in driving these changes, which are substantially mediated by NMDARs.

## 4 Discussion

The goal of this study was to examine the long-lasting effects of traumatic stress on structural plasticity in an MeApv-VmHvl pathway. Using the mGRASP method we found that foot shock during late adolescence/early adulthood increases synapse number between the MeApv and VmHvl as indicated by an increase in the number of mGRASP puncta in the VmHvl of animals that received foot shock, compared to those that did not. Furthermore, we demonstrated that chemogenetic inhibition of excitatory neurons in the MeApv suppressed the increase in mGRASP puncta after foot shock. Notably, these effects were largely restricted to synapses on the shaft of the VmHvl dendrites, while leaving total spine number unchanged. Moreover, systemic administration of an NMDAR antagonist before foot shock produced a similar effect to chemogenetic inhibition of MeApv neurons, strongly suggesting that the structural plasticity in the MeApv-VmHvl pathway after foot shock is NMDAR-dependent. These findings demonstrate a novel mechanism for the effects of traumatic stress on structural plasticity.

### 4.1 Morphological characterization of structural plasticity

The localization of synaptic structural changes to the distal dendritic shaft as opposed to the distal spines is intriguing. Dendritic shafts and spines serve different functions and capacities for synaptic integration. Shaft synapses may contribute more to global dendritic signaling, while spine synapses are often implicated in localized, compartmentalized processing (Harnett et al., 2012). Mature spines have a bulbous head connected to the dendritic shaft by a thinner neck. These morphological traits affect the influence of spines on neuronal signaling; the thin neck of the spine restricts molecular diffusion into and out of the shaft (Svoboda et al., 1996; Bloodgood and Sabatini, 2007; Adrian et al., 2014). Functionally, this means that spine synapses have a modulatory effect on neuronal signaling, while shaft synapses are situated to drive neuronal firing. Our finding of a marked increase in distal shaft synapses following traumatic stress might suggest a broader influence on neural firing, which could result in more generalized behavioral effects.

Dendritic spines, which are the primary sites for excitatory synaptic input, can be categorized based on their distance from the cell body into proximal and distal spines (Perez-Rando et al., 2017). Their location along the dendrite not only influences their physiological properties but also their stability and turnover rates (Jibiki et al., 1978; Vu and Krasne, 1992; Nonaka and Hayashi, 2009). Proximal dendritic spines, being closer to the cell body, are generally considered more stable than their distal counterparts. This stability is thought to be linked to their role in maintaining consistent and robust synaptic connections essential for basic cellular functions. Interestingly, NMDARs on proximal spines have been found to produce stronger long-term potentiation (LTP) and structural plasticity than NMDARs on distal spines (Parvez et al., 2010; Ferreira et al., 2020).

In our study, the notable alterations observed in response to foot shock were concentrated in these proximal, traditionally more stable, dendritic spines. The fact that these normally resilient spines showed significant changes underscores the profound impact of traumatic experiences on neuronal architecture. Our finding that chronic inhibition curtailed these alterations further spotlights the importance of understanding and potentially targeting the mechanisms underlying proximal spine plasticity. Future research should be directed at answering these questions.

### 4.2 Traumatic stress-induced aggression

Previously, we demonstrated that traumatic stress induces a dramatic increase in aggression through synaptic potentiation of MeApv-VmHvl synapses (Nordman et al., 2020a,b; Nordman, 2021). The observed increase in aggression lasted up to 7 days following foot shock, which we define as long-lasting. Potentiation of existing MeApv-VmHvl synapses is not sufficient to explain such a long-lasting increase, as the timescale of synaptic potentiation is minutes to hours, not days (Huang and Kandel, 1994). In contrast, the formation of new synapses (i.e., structural plasticity), can last for days or longer (Bernardinelli et al., 2014). Therefore, it follows that the long-lasting traumatic stress-induced aggression and potentiation that we reported previously was likely the result of structural plasticity within the MeApv-VmHvl pathway.

Further supporting this conclusion, we demonstrated that MeApv-VmHvl synapses could only be depotentiated within the first 24 h after traumatic stress; when applied after the 24-h mark our optogenetic depotentiation protocol had no effect on aggression (Nordman et al., 2020b). This finding suggests that more permanent and stable changes occurred after 24 h that were not easily reversed through our depotentiation protocol. Future studies should investigate if a stronger depotentiating protocol or synapse elimination method could suppress or reverse the long-lasting increase in aggression and synaptogenesis after foot shock. Exciting new optogenetic methods have been developed that may be useful in this context (Hayashi-Takagi et al., 2015; Moda-Sava et al., 2019).

### 4.3 Extrasynaptic NMDARs and synaptic plasticity after foot shock

The influential role of NMDARs in synaptic and structural plasticity is well-established. However, the emerging importance of extrasynaptic NMDARs in this arena has become a focal point of recent research (Emnett et al., 2013). Our observations indicate that foot shock primarily impacts the dendritic shafts within the MeApv-VmHvl pathway. As dendritic shafts are recognized centers for extrasynaptic NMDAR activity (Oikonomou et al., 2012), a discussion of the specific functions these potential receptors have in foot shock-induced structural plasticity is justified.

Our findings indicate that application of MK-801, an antagonist that affects both synaptic and extrasynaptic NMDARs (Song et al., 2018), curtails the increase in shaft and spine synapses along with aggression following foot shock (Nordman et al., 2022). Such a reduction underscores the potential role of extrasynaptic receptors in regulating the synaptic and structural shifts prompted by traumatic events. Corroborating this notion, our prior research highlighted that memantine, a specific extrasynaptic NMDAR antagonist (Emnett et al., 2013), diminishes aggression induced by traumatic stress (Nordman, 2021; Bartsch and Nordman, 2022; Nordman et al., 2022), likely by targeting the MeApv-VmHvl pathway.

Therefore, it is plausible that foot shock activates extrasynaptic NMDARs, initiating a sequence of internal cellular activities that result in structural modifications, especially within dendritic shafts. This could be a natural adaptive response of the neural system to chronic external threats, though it may lead to detrimental changes if the stimuli are traumatic, like foot shocks. Indeed, extrasynaptic NMDARs have previously been linked to mood and anxiety disorders stemming from enduring social defeat stress (Tse et al., 2019). Developing more specialized extrasynaptic NMDAR-targeted interventions could be a promising avenue for future research.

### 4.4 Problems and potential pitfalls

In our study, we acknowledge certain limitations that need to be addressed for a comprehensive understanding of the impact of traumatic stress on MeApv-VmHvl structural plasticity.

First, the mGRASP method we employed did not allow for within subject comparisons. The nature of our approach was such that the tissue needed to be collected following the manipulation, so that we were unable to measure real time changes in synapse number (Lee et al., 2023). Future studies could combine the mGRASP technique with *in vivo* two-photon microscopy to longitudinally monitor synapse formation within a single subject. This has been done with the similar eGRASP system to examine structural changes in a hippocampus circuit after fear induction (Lee et al., 2023). However, there are significant technical hurdles to imaging tissue as deep as the VmHvl, along with the high potential of damage to the mGRASP puncta using implants such as a GRIN lens (Miller et al., 2019). These factors precluded us from using this method, but newer alternatives may be developed.

Second, it is possible that only a subset of MeA-VmH synapses is responsible for the structural plasticity and behavioral changes associated with traumatic stress. Our current methodology does not allow us to investigate context-dependent expression of mGRASP. Future studies could address this by combining mGRASP and the context-dependent c-Fos-rtTa3G expression system, as in recent studies (Choi et al., 2018, 2021; Murthy et al., 2022; Lee et al., 2023).

Third, our study does not directly address cell type in the MeApv-VmHvl pathway. Specifically, it is unknown to us whether the increase in mGRASP occurs on excitatory or inhibitory cells. This could be remedied using cre-dependent post-mGRASP or a similar system in animals expressing cre-recombinase in various established VmHvl cell types (Kim et al., 2019). Pursuing such approaches in future research will be crucial for direct and precise mapping of the underlying circuitry.

Finally, the design of our study does not allow for determination of causality. An increase in mGRASP puncta following foot shock does necessarily indicate that structural plasticity is responsible for traumatic stress-induced aggression increase. Newer methods may be able to address this question (Moda-Sava et al., 2019).

## 5 Summary

This study underscores the significance of structural plasticity in traumatic stress. Per our previous studies, these modifications likely drive maladaptive behavioral changes. Importantly, these neural adaptations are driven by glutamatergic signaling and an NMDAR-dependent mechanism, possibly leading to changes in dendritic shaft plasticity. This nuanced relationship between dendritic structure and receptor activity could offer a neural blueprint for trauma that paves the way for innovative and transformative therapeutic interventions.

## Data availability statement

The original contributions presented in the study are included in the article/Supplementary material, further inquiries can be directed to the corresponding author.

## Ethics statement

The animal study was approved by Animal Care and Use Committee of Southern Illinois University School of Medicine. The study was conducted in accordance with the local legislation and institutional requirements.

## Author contributions

JN: Conceptualization, Data curation, Formal analysis, Funding acquisition, Investigation, Methodology, Project administration, Resources, Software, Supervision, Validation, Visualization, Writing – original draft, Writing – review and editing. CB: Data curation, Formal analysis, Funding acquisition, Investigation, Methodology, Validation, Visualization, Writing – original draft, Writing – review and editing. JJ: Conceptualization, Data curation, Formal analysis, Investigation, Methodology, Resources, Software, Validation, Visualization, Writing – original draft, Writing – review and editing. NM: Data curation, Investigation, Methodology, Validation, Visualization, Writing – review and editing. EQ: Data curation, Formal analysis, Funding acquisition, Investigation, Validation, Visualization, Writing – review and editing. MS: Data curation, Formal analysis, Investigation, Methodology, Validation, Visualization, Writing – review and editing. OC: Data curation, Formal analysis, Methodology, Validation, Visualization, Writing – review and editing. SA: Validation, Visualization, Writing – review and editing, Data curation, Formal analysis, Funding acquisition, Methodology.
