# Supplementary figures and images for: Visualizing traumatic stress-induced structural plasticity in a medial amygdala pathway using mGRASP
Source: Front Mol Neurosci. 2023 Nov 30;16:1313635. doi: 10.3389/fnmol.2023.1313635 (PMC10720331; doi:10.3389/fnmol.2023.1313635)

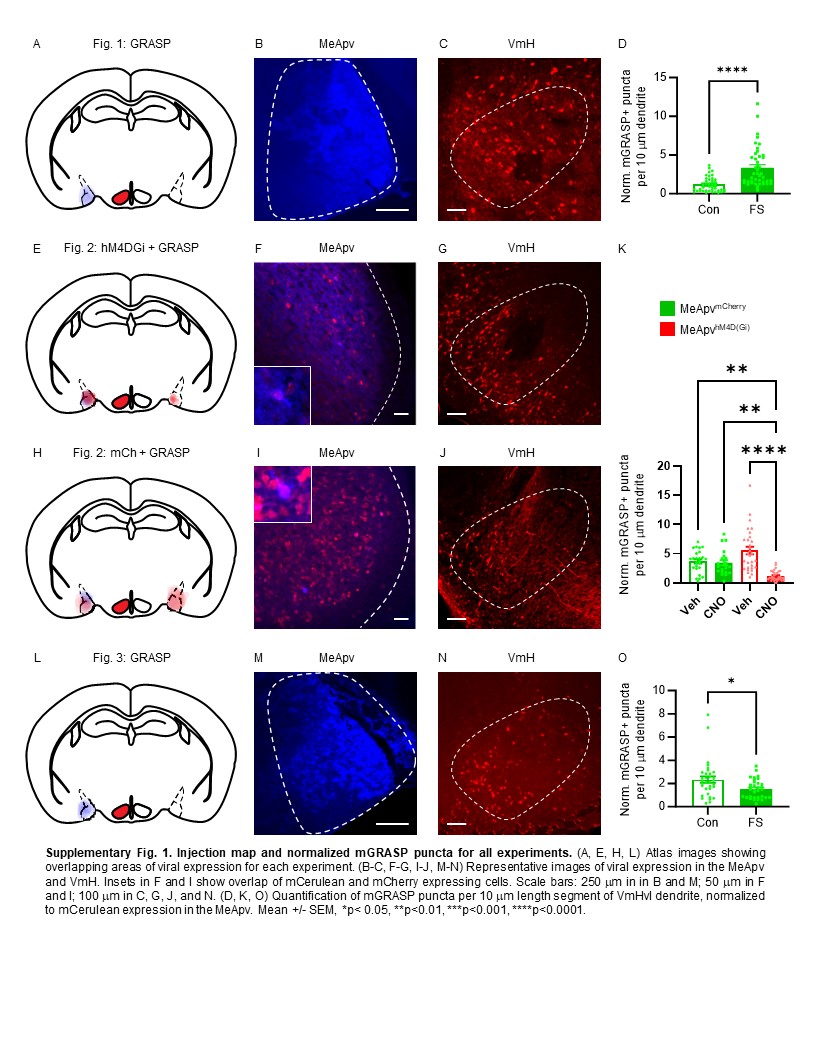

Supplement: Supplementary file 1 [file Image_1.JPEG]

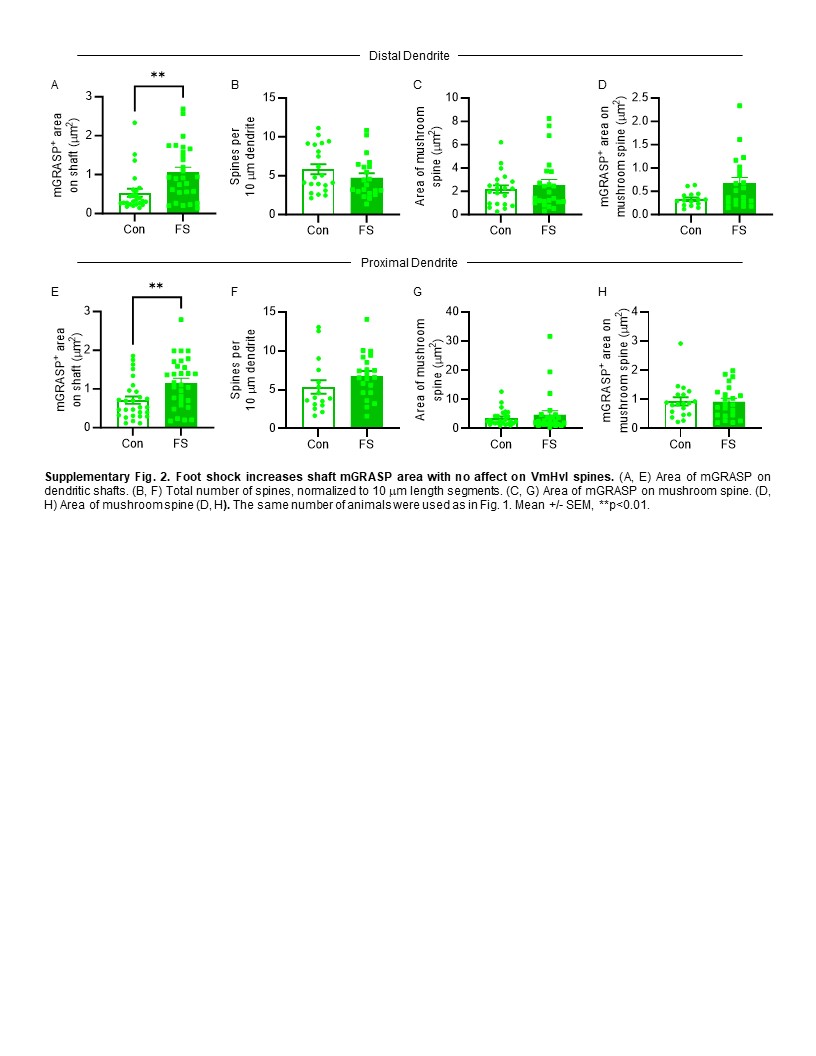

Supplement: Supplementary file 2 [file Image_2.JPEG]

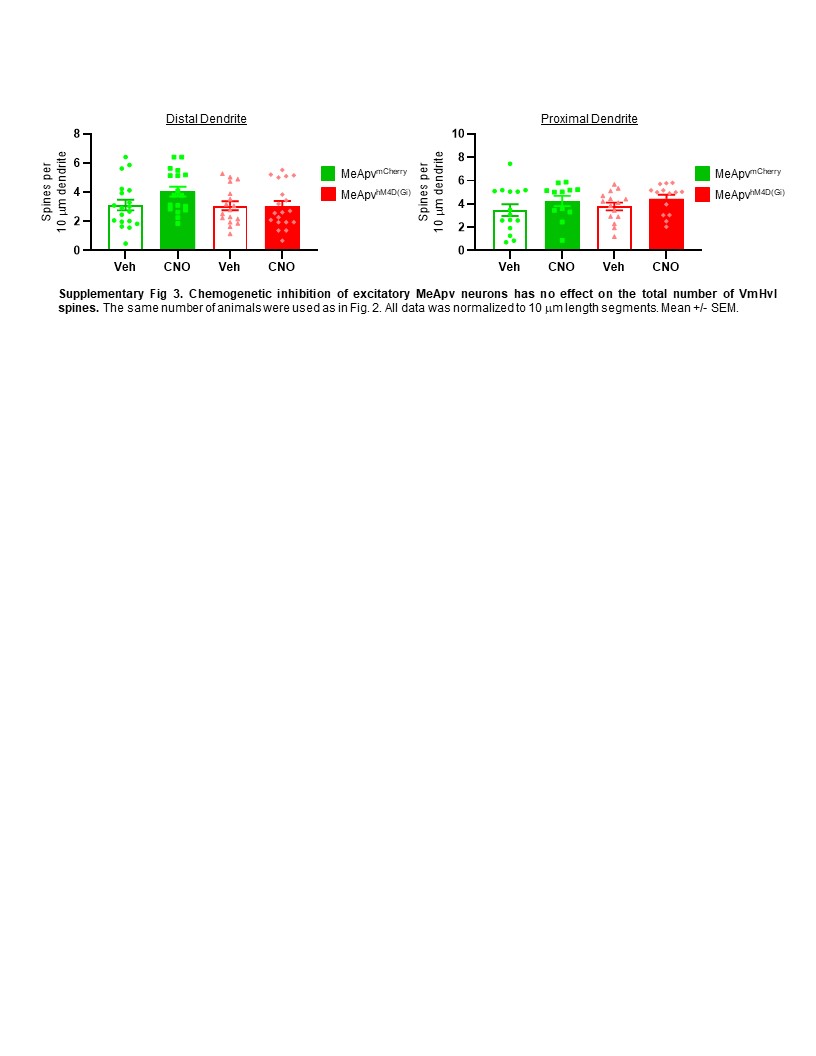

Supplement: Supplementary file 3 [file Image_3.JPEG]

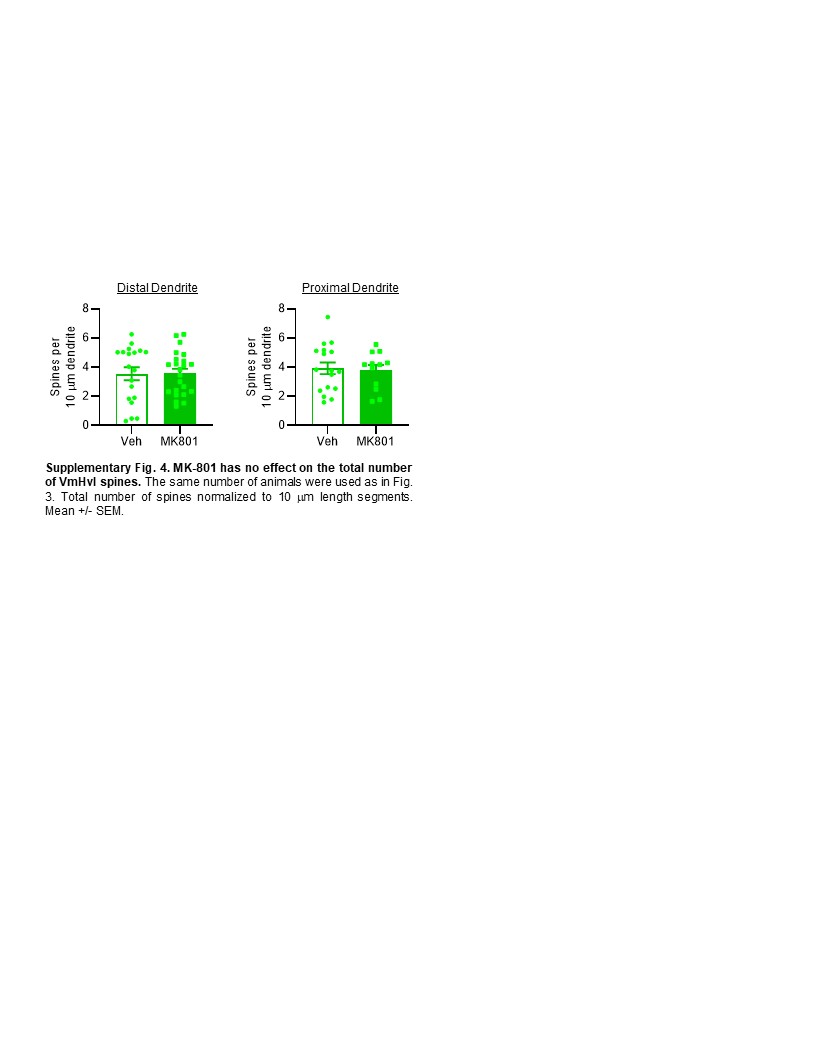

Supplement: Supplementary file 4 [file Image_4.JPEG]
